# Post-floral Erection of Stalks Provides Insight into the Evolution of Fruit Orientation and Its Effects on Seed Dispersal

**DOI:** 10.1038/srep20146

**Published:** 2016-02-02

**Authors:** Yang Niu, Zhuo Zhou, Wen Sha, Hang Sun

**Affiliations:** 1 Key Laboratory for Plant Diversity and Biogeography of East Asia, Kunming Institute of Botany, Chinese Academy of Sciences, 132 Lanhei Road, 650201, Kunming, Yunnan, China

## Abstract

That stalks reorient after flowering to face upwards is a common phenomenon in many flowering plants, indicating the potential importance of fruit orientation on seed dispersal. But this idea has not been subject to an empirical test. We examined this hypothesis by analysing the evolutionary correlation between fruit orientation and other characters and by investigating the effects of fruit orientation on seed dispersal. We found that 1) in a sub-alpine plant community, upward fruit orientation strongly correlates with fruits that act as seed containers, which are often of dry type and are dispersed by non-animal vectors; 2) as exemplified by the Campanulaceae *s. str.*, fruit orientation strongly correlates with dehiscence position. Upwardly-oriented capsules dehisce at the apex, whereas pendent ones dehisce at the base, in both cases ensuring that seeds are released from an upright position; 3) in manipulation experiments on *Silene chungtienensis*, upward fruits (the natural state) exhibit much greater dispersal distances and more dispersive pattern than pendent ones, and have a more even distribution of dispersal direction than horizontal ones. Our results suggest that fruit orientation may have important function in seed dispersal, which may be the reason why the phenomenon that stalk erection after flowering occurs widely.

The fruit is a characteristic structure in flowering plants, and its character affect the way of seed dispersal. It is well known that specific morphological features may enhance dispersal by the relevant agents^1^. Other fruit characters, such as size^2^,^3^, colour^4^,^5^,^6^, scent^6^,^7^,^8^ and quality^9^, have also been shown to have significant effects on the dispersal process. However, most studies have focused on how those fruits act as diaspores (dispersal units), e.g., how berries, nut and achenes are dispersed. There are numerous fruits that do not separate from their mother plants, acting solely as seed containers (e.g., most capsules and follicles). For these plants, the effects of fruit characters on seed dispersal have received much less attention. One of these neglected characters is fruit orientation, and there is widespread clue indicating that this may have potential effects on plant fitness.

A striking feature of fruits that act as seed containers is their upward orientation. Many plants whose flowers are not upwardly orientated erect their stalks after flowering, thus holding the fruits in an upward orientation^1^, (e.g., Fig. 1a). In a study on *Anisodus luridus* (Solanaceae), Wang *et al.*^10^ suggested that the erect stalks hold rainwater within the persistent calyx, providing better conditions for seed development. However, this mechanism may not be widely applicable to other plants that show similar phenomenon. For most plants, it is plausible to speculate that this phenomenon may be a mechanism for improving seed dispersal^1^,^11^,^12^, given that erecting the stalks may raise fruits to a position suitable for dispersal. Surprisingly, however, we have found no study testing this hypothesis to date. Whether or not the hypothesis is a reasonable one, we lack knowledge about the way in which fruit orientation may affect seed dispersal.

To test this hypothesis, the first question we asked was: do some taxa exhibit upward fruit or stalk erection more frequently than others? An upward fruit orientation is likely to be more important for plants that produce containers, which require a suitable position from which to release seeds. We would therefore expect to find a correlation between fruit orientation and the function of the fruit, i.e., whether it acts as a diaspore or as a seed container. Furthermore, fruit orientation may also correlates to other fruit characters, such as fruit type and dispersal mechanisms. Erecting the stalks after flowering had been more frequently reported in capsule- and follicle-bearing taxa^1^,^12^, but a more rigorous investigation is needed to get a more comprehensive understanding of the distribution pattern of this phenomenon.

Secondly, we asked why there are a number of plants that keep their containers pendent (e.g., Fig. 1b) and what is the evolutionary pattern of fruit orientation. To our knowledge, the fruit types in the Campanulaceae *s. str.* are dominated by capsules, but the orientation of the fruits varies among them remarkably, being either pendent or upward. Furthermore, the position of dehiscence in the capsule also varies in this family^13^, from base to apex. Obviously, an upright release position can only be ensured when upward-oriented fruits dehisce at the apex rather than at the base. We would therefore expect a correlation between fruit orientation and dehiscence position in these plants.

Finally, we asked how fruit orientation could affect seed dispersal. We anticipate that an upward fruit may have better control over the release of seed relative to other fruit orientations, thus promoting dispersal. These effects may be achieved through dispersal distance, dispersal direction and/or the general pattern of seed distribution. All of these dispersal parameters may have potential influence on plant fitness through their effects on the fate of seeds^14^.

To answer these questions, we first investigated the distribution pattern of fruit orientation and its correlation with other characters in a natural plant community, and then used a model system, the bluebell family, to investigate the evolutionary pattern of fruit orientation and dehiscence position. Finally, we examined the effects of fruit orientation on seed dispersal by conducting phenotypic manipulation experiments in the field, using *Silene chungtienensis* (Caryophyllaceae) as a model system.

## Results

### Evolution of fruit orientation and associated characters

#### Fruit characters in a sub-alpine plant community

In the community we studied in SW China, 80 species from 66 genera and 34 families were included in the analysis. The distribution of the fruit characters is shown in Supplementary Fig. S1. Among these plants, both the upward-oriented fruit (53 cases) and the stalk erection phenomenon (33 cases, characterized by a non-upward-oriented flower followed by an upward fruit) occur more frequently in fruits that act as seed containers, in taxa with dry fruits and are dispersed by non-animal vectors (Table 1 and Supplementary Table S1).

Fruit orientation and all three other characters (fruit function, fruit type and dispersal vector, analysed separately) evolves in a correlated way, given that the dependent evolutionary model fit the data better (BayesTraits^15^, Table 1). Specifically, an upward fruit orientation strongly associates with fruits that act as containers, dry fruits (capsules, follicles and achenes) and non-animal dispersal vector (wind). In contrast, a pendent fruit orientation associates with fruits that act as diaspora, fleshy fruits and animal dispersal vector. We got a similar association between the stalk erection phenomenon and these characters we concerned (Supplementary Table S1). However, there are some exceptions in the clade of Campanulaceae, where pendent capsules occur without the stalk erection phenomenon (Supplementary Fig. S1). We also noticed pendent orientation in a few leguminous dry fruits.

When the three predictors (fruit function, fruit type and dispersal vector) were considered together in the Bayesian mixed model (MCMCglmm^16^, with phylogeny as a random effect), only fruit function had a significant influence on fruit orientation (Table 2) and only dispersal vector had a significant influence on the stalk erection phenomenon (Supplementary Table S2). These results are possibly because there are internal correlations among these predictors (Pearsman correlation *P* < 0.001 for all predictor pairs), causing collinearity. When testing each predictor separately, all of them had a significant influence (pMCMC < 0.001 for all tests).

#### Fruit orientation and dehiscence position in the Campanulaceae s. str

The Bayesian Inference tree based on four plastid markers with posterior probabilities is shown in Fig. 2. Fruit type, orientation and dehiscence position varies among taxa in the bluebell family. Most of the taxa with capsules have upward-orientated fruits, whereas all of the taxa with berries have pendent fruits. However, it is worth noting that some members of *Asyneuma* (e.g., *A. trichocalycium*), many members of *Campanula* and all members of *Adenophora* and *Hanabusaya* produce pendent rather than upward capsules. We further investigated why pendent capsules occur in these plants, and a strong evolutionary correlation between fruit orientation and dehiscence position was discovered (BayesTraits^15^, likelihood ratio value 77.52, *d.f.* = 4, *P* < 0.001). Specifically, almost all species with upward capsules have an apical dehiscence position, whereas species with pendent capsules have a basal dehiscence position. Likelihood (Fig. 2) and parsimony (Supplementary Fig. S2) analysis of character evolution showed identical result, with the upward capsule (proportional likelihood probability as 0.613) and the apical dehiscence position (0.681) most likely to be the ancestral state in the Campanulaceae *s. str*. The pendent capsules and basal dehiscence position have evolved independently several times in this family (especially in *Campanula*). We noticed that *Merciera tenuifolia* (with indehiscent fruit) does not obey the correlation we mentioned above. Interestingly, however, this species produces few large seeds rather than numerous small ones, and is thus quite different from other members of the family in the dispersal mechanism.

### The effects of fruit orientation on seed dispersal in *Silene chungtienensis*

#### Distance

Seeds from the three groups (upward, pendent and horizontal, Fig. 3) showed different distributions of dispersal distance (Fig. 4a). One-way ANOVA showed that the orientation of the capsule has a significant effect on the mean distance of seed dispersal (*F*_*2,43*_ = 89.789, *P* < 0.001). The distance in the pendent group was much shorter than that in the other two groups. There was no significant difference in mean dispersal distance between upward and horizontal groups (Fig. 4b).

#### Direction

The direction of seed dispersal showed different patterns among the three groups (Table 3). Only the upward group had an almost equal proportion of seeds deposited in each of the four quadrants (e.g., Supplementary Fig. S3a). For the pendent group, quadrant I received a significantly higher proportion of seeds than the other three quadrants (e.g., Supplementary Fig. S3b). For the horizontal group, the two southern quadrants (I and IV) had a much higher proportion of the seeds than the two northern ones (II and III, e.g., Supplementary Fig. S3c), possibly due to the direction of the opening and the prevailing wind direction during the study season.

#### General distribution pattern

All (15) samples of the pendent group showed a strongly clustered pattern of seed distribution. In contrast, only five samples (out of 15) from the upward group and nine samples (out of 16) from the horizontal group showed a clustered pattern; other samples showed a random pattern of distribution. When seed positions from samples within each group were pooled, all three groups showed a clustered distribution; however, the intensity of clustering differed among them. The pendent group showed the strongest clustering pattern (with a most steep curve, Supplementary Fig. S4), implying a high seed density at particular scales.

## Discussion

In the present study, the data of fruit traits were obtained from a sub-alpine community in SW China, but they covered many representative taxa that widely distributed in the temperate area in the Northern Hemisphere. In this community, over half of the species exhibit upward-oriented fruit. There are strong evolutionary correlations between fruit orientation and the three other characters, i.e. fruit function, fruit type and dispersal vector. When considered these characters together, fruit function is a better predictor to fruit orientation. The phenomenon that erect stalks after flowering also strongly correlates with the these characters, it frequently occurs in fruits that act as containers, fruits of type and are dispersed by non-animal vectors. Among these characters, dispersal vector performs better in predicting the presence of this phenomenon. This may explain why a few taxa with diaspores fruits (achenes) that are dispersed by wind (e.g., *Clematis akebioides* in this study and *Pulsatilla cernua* in Huang *et al.*^17^) also exhibit this phenomenon. The relatively heavy weight of fleshy fruit could be a simple explanation for why most of them are pendent, although this habit may also be more attractive to frugivorous animals. Similarly, legumes with large seeds also exhibit pendent orientation. However, the lighter weight of most dry fruits does not necessarily explain why many of them adopt an upward fruit orientation, especially in those species whose flowers are non-upward. These results suggest that a specific fruit orientation may be of particular functional importance for these plants.

Seeds from capsules and follicles are usually small and have no features to facilitate dispersal by specific vectors. These seeds could be indirectly dispersed by wind^12^, a process that has been defined as anemoballism by van der Pijl^1^, jactitation by Ridley^18^ and the censer mechanism by Bansal and Sen^19^. Although the mechanism of this dispersal type has received quite limited attention, van der Pijl^1^ noted that “*… Many Campanulaceae are comparable* (to papaver) *and also have pores at the top of erect capsules and at the base of hanging ones, preventing the seeds from simply falling out*”. This correlation between fruit orientation and dehiscence position is the very one that we found in the Campanulaceae *s. str.* in this study. The dehiscence position is always in the upper part in upward capsules, whereas it is at the base in pendent ones, in both cases ensuring that seeds are released from the upright position. Our analysis of character evolution showed that the ancestral condition was likely to be an upward capsule accompanied by apical dehiscence, and that the pendent capsule combined with basal dehiscence independently evolved several times in this family.

When gathering information on fruit characters, we found that taxonomists had already observed the variation in dehiscence position that occurs in the Campanulaceae^13^ and had even used it as a key character in some taxa (e.g., *Campanula*^20^), but fruit orientation has seldom been noted or described. In the genus *Campanula*, we found that closely related species may have different dehiscence positions. This suggest that dehiscence position, which strongly co-varies with fruit orientation, is not a conservative enough feature to be used as a key character for classification at the subgenus-level (this was also implied by other studies, such as Mansion *et al.*^21^). It is possible that the capsule orientation and dehiscence position are strongly genetically correlated. Alternatively, these two traits may have been under strong selective pressure, as they may have influence on plants’ reproduction through their effects on seed dispersal.

How might fruit orientation affect seed dispersal? Results from the phenotypic manipulation study showed that seeds released from upward fruits dispersed over a greater distance, more evenly in direction and more dispersive than those from pendent and horizontal fruits, a finding consistent with our predictions. Although a greater dispersal distance does not always ensures greater fitness^22^, many plants potentially get advantage by disperse seeds farther or get lower seed density (often as a covariant) because their offspring may reach better sites through avoiding natural enemies or detrimental sibling interactions (reviewed by Willson & Traveset^14^; Fenner & Thompson^22^). As a typical wind-ballist^1^, *Silene chungtienensis* has seeds that are small and can be released when wind sways the capsule. For plants with this dispersal syndrome, an upright release position is essential to control the timing of seed release (i.e. seeds are released when wind sways the fruits), promoting greater dispersal distances. Furthermore, after fruits swing in one direction the stalk usually rebounds in the opposite direction (see Weiblen & Thomson^23^), which may explain why the upward group has a more random and dispersive seed distribution. In the pendent group, on the other hand, seeds fall out without any effective means of control, resulting in much shorter dispersal distances and intensely clustered distributions. In the case of the horizontal group, more seeds are dispersed in a specific direction because of the direction of the mouth opening (and the wind), although their dispersal distance is similar to that of the upward group. We concede that although the differences in dispersal parameters are significant, we need further works to investigate whether they could translate into differences in plant fitness (for example, see Donohue^24^ and Wender *et al.*^25^ for the effects of seed density at a similar scale as this study).

Plant height has been considered to be an important factor affecting seed dispersal distance^26^. It is possible that erect stalks may raise fruits to higher release positions, promoting dispersal. However, this phenomenon *per se* may have only a limited effect on release height; it is the accompanying marked elongation of the stalks that raises the fruits to higher positions in many cases^11^. In this study, we controlled the effects of plant height and found that fruit orientation *per se* had significant effects on seed dispersal by *Silene chungtienensis*. In order to monitor the movement of seed in a limited space, the seed release height in our experiments was lower than it would be under natural conditions. We appreciate that the dispersal distance is likely to be greater when this species releases its seeds under natural conditions, but we do not believe that this would alter our qualitative conclusions.

The effects of flower orientation on plant reproductive success have been of interest since the time of Sprengel^27^. However, fruit orientation has received much less attention, even though seed dispersal is also an essential component of a plant’s life history. It has been recognized that the requirements of floral orientation for pollination may response to both biotic (pollination accuracy^28^,^29^) and abiotic (rain and/or UV-radiation damage^30^,^31^) factors. However, these requirements may not be consistent with the requirements for optimal seed dispersal, and this may be the fundamental reason why the erection of stalks after flowering occurs widely.

## Methods

### Orientation and other associated fruit characters in a sub-alpine plant community

To answer the question of whether upward-oriented fruit and stalk erection occur more frequently in some taxa than in others, we investigated the distribution of several fruit characters in a sub-alpine plant community in Shangri-La Alpine Botanical Garden (27°54′N, 99°38′E, elevation 3355 m a.s.l.). This is a natural botanical garden, located in the biodiversity hotspot region in SW China, which was established to protect natural vegetation from grazing. We recorded the common plant species in meadow and shrub habitats, and then constructed a phylogenetic tree by grafting the genera present in the study area onto a backbone phylogenetic hypothesis using the online program Phylomatic^32^. The backbone of the tree was the Phylomatic tree version R20120829, with the reliability of phylogenetic relationships among the family and the support values of the clade all based on Angiosperm Phylogeny Group’s system^33^. The weakly supported clade collapsed in the Phylomatic tree. Branch lengths in the tree were adjusted to match clade age estimates reported by Wikström^34^ using the BLADJ algorithm implemented in the software Phylocom^35^. Relationships among the genera within a given family, and among species within a genera were treated as polytomies, as the scarcity of comprehensive time-calibrated phylogenies within families.

For each species, we assigned states for the following four characters: fruit function (diaspore or seed container), fruit orientation (upward or non-upward), fruit type (dry or fleshy) and expected dispersal vector (animal or non-animal). We also noted whether stalk erection took place after flowering (present or absent), characterized by a non-upward-oriented flower/inflorescence followed by an upward fruit/infructescences. The information involved were obtained either from direct observation or from the literature (i.e. Flora of China^36^). Berries, drupes and other berry-like fruit were classified as fleshy fruit, and capsules, follicles, achenes and other non-fleshy fruit were classified as dry fruit. The diaspores that were edible (endozoochores) or equipped with barbs (epizoochores) were regarded as being animal-dispersed. Those diaspores with wings, hairs or pappi (anemochores), and that had no special dispersal device and were enclosed in dehiscent containers (anemoballists) and autochores were classed as non-animal dispersed^1^,^12^. Note that although the seeds of *Viola delavayi* have elaiosomes that facilitate ant dispersal, they are autochores in the primary dispersal process.

### The evolution of fruit orientation and dehiscence position in the Campanulaceae s. str

Because the fruit orientation (upward or pendent) and the dehiscence position (at the apex or the base) varies among taxa within the Campanulaceae *s. str.*^13^,^20^, we used this family as a model to investigate the potential correlation between these two characters and their evolution. A total of 82 taxa representing all three major clades of the Campanulaceae *s. str.* (the platycodonoid, wahlenbergioids and campanuloids clade; see Haberle *et al.*^37^) were selected for the phylogenetic analysis. We used four chloroplast markers (*atpB*, *matK*, *rbcL*, *trnL-trnF*) to reconstruct the relationships within the family, building on previous phylogenetic studies in the family level carried out by Haberle *et al.*^37^ and Roquet *et al.*^38^ We did not include the nuclear genes for two reasons: (1) the only nuclear gene used to reconstruct the phylogeney at the family level of Campanulaceae was nuclear ribosomal ITS regions^39^, however, aligning ITS sequences across genera in the Campanulaceae is difficult due to a high level of ambiguities in the ITS1 and ITS2 regions^39^,^40^. (2) the phylogenetic tree based on nrITS^39^ corroborated to the topology only based on plastid markers^37^,^38^,^41^, the three major clades and many subclades within the family were identical according to nuclear and plastid genes. We revised the names of a few taxa (from the platycodonoid group) according to the recent study by Wang *et al.*^42^ Sequences from five Chinese *Campanula* species (*C. modesta*, *C. aristata*, *C. pallida*, *C. cana* and *C. nakaoi*) that showed variation in dehiscence position and fruit orientation were newly generated in the current study (Supplementary Table S3), while all other sequences were obtained from GenBank (Supplementary Table S4). We inferred phylogenetic relationships from the combined plastid data set using Bayesian analyses. Before the analysis, the appreciate nucleotide substitution model for our data was determined by jModelTest 2.0^43^. The best model selected under the Akaike Information Criterion^44^ was TVM + I + G (with nst = 6, rates = gamma). Bayesian inference (BI) was performed in the program MrBayes 3.1^45^ based on the previously determined model, the Markov chain Monte Carlo (MCMC) algorithm was run for 2,000,000 generations with one cold and three heated chains, starting from random trees. Trees were sampled every 100 generations. The first 25% of runs were discarded as burn-in. A majority rule consensus of the remaining trees from the four runs was produced in PAUP* 4.0^46^ and used as the Bayesian inference tree with posterior probabilities (PP).

Analysis of character evolution was based on the topology of Bayesian inference tree with branch lengths. To make the results more reliable, we performed our reconstruction using both the maximum parsimony and maximum likelihood method conducted in Mesquite 4.2^47^. In the parsimony method, the character states were treated as unordered. We employed the Markov k-state1 (Mk1) parameter model of evolution for the likelihood reconstruction, where all changes are equally probable. The character states for fruit orientation were pendent, upward and unknown. The character states for fruit dehiscence position were base, apex, berry and unknown, indehiscent or no specific dehiscence position.

To collect the character information, we consulted descriptions and botanical illustrations in publications (e.g., monographs and local Flora), and checked specimens in the herbaria (KUN, PE and online data sources). For several species that detailed information (especially for fruit orientation) cannot be obtained by these ways, we searched for photos or illustrations on the internet using Google Image and then checked the identification reliability according to specimens or descriptions. Only those images with reliable identification were used in analysis. Direct observations in the field were also an important source of data, especially for taxa from China. The character information we used and their sources were listed in Supplementary Table S5.

### Tests for characters correlations

We used a likelihood approach to test the correlated evolution among fruit orientation and other fruit characters we concerned in the sub-alpine community and the Campanulaceae *s. str*., using BayesTriats (V2)^15^. The BayesDiscrete module was used to examine correlated evolution between pairs of discrete binary traits (as mentioned above) by comparing the fit (log likelihood) of two continuous-time MCMC model (independent vs. dependent). The independent model assumes the two traits evolve independently, and has four transition parameters. The dependent model assumes the traits are correlated and the transition rate of one trait depends on the state of the other, and thus has eight parameters. A support for correlated evolution of two traits can be tested by comparing 2[log-likelihood (better fitting model) – log-likelihood (worse fitting model)] to a *χ*^*2*^ distribution with four degrees of freedom (omnibus test^48^). The α level was adjusted by Bonferroni correction in the community analysis, as we conducted multiple tests. Note that only binary traits were used in these analyses. In the Campanulaceae, when information was unavailable (3 species) and when the fruits are berry (4 species), indehiscent (1 species) or have no specific dehiscence position (2 species), the relevant character states were treated as missing values. For data from the sub-alpine community, we also examined how the stalk erection phenomenon was related to fruit function, fruit type and dispersal vector using Bayesian mixed models (with phylogeny as a random effect) as implemented in the R package MCMCglmm^16^, running 110,000 iterations with a burn in period of 10,000 and a thinning interval of 100 and using uninformative priors.

### The effect of fruit orientation on seed dispersal

#### Study system

**Figure 1:**
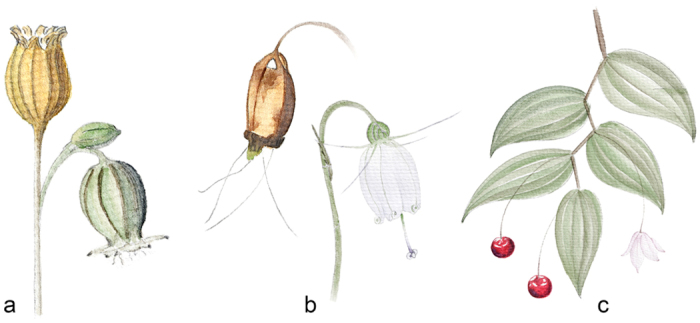
Examples showing the orientation of flowers and fruits. Pendent flowers are succeeded by upward capsules that dehisce at the apex in *Silene chungtienensis* (Caryophyllaceae) (**a**); pendent flowers and pendent capsules that dehisce at the base in *Adenophora capillaris* subsp. *leptosepala* (Campanulaceae) (**b**); and pendent flowers and pendent berries in *Streptopus simplex* (Liliaceae) (**c**). Illustrated by Wen Sha.

**Figure 2:**
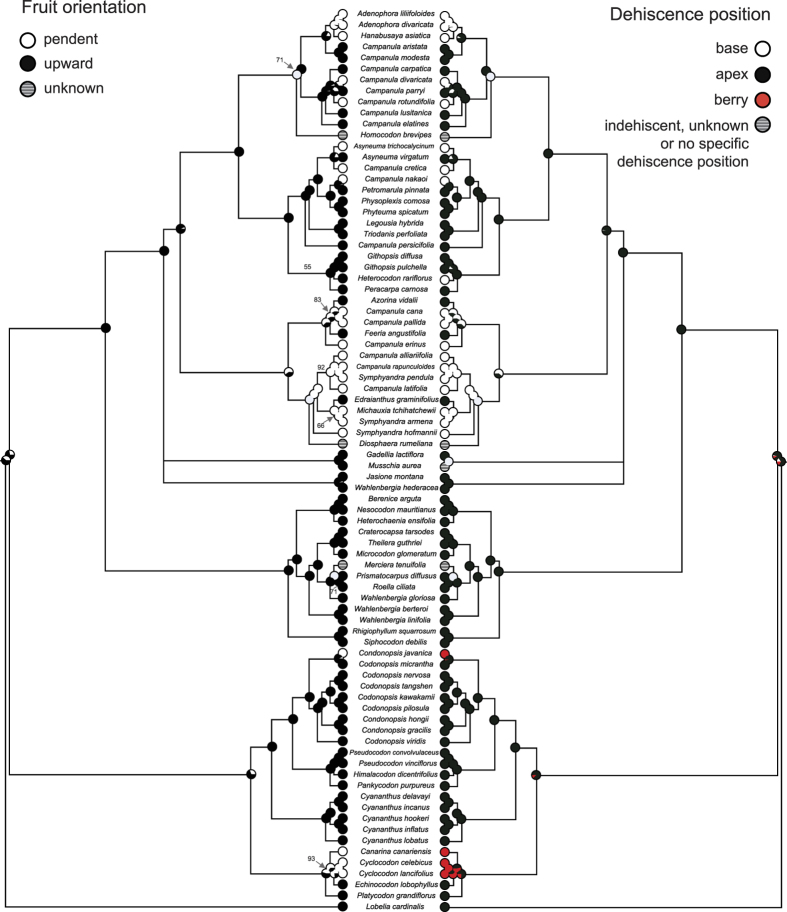
Likelihood reconstruction of ancestral states of fruit orientation (left) and dehiscence position (right), and visualized correlation between them in plants of the bluebell family. The Bayesian inference tree was reconstructed based on four chloroplast markers (*atpB, matK, rbcL, trnL-trnF*), using 82 taxa representing all the three major clades in the Campanulaceae *s. str*. Proportional likelihoods of ancestral states at nodes are represented by pie charts. Bayesian posterior probabilities < 0.95 were indicated with corresponding number on related node, while all other nodes without indication were strongly supported node (i.e., Bayesian posterior probabilities ≥0.95).

**Figure 3:**
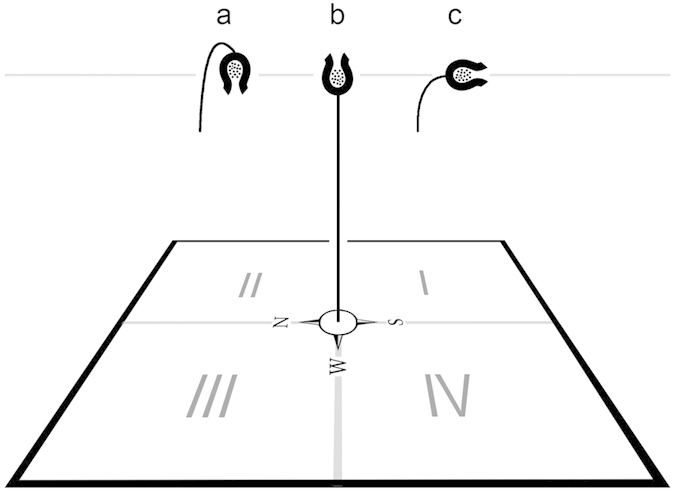
The sticky trap device used in manipulation experiments for studying the effects of fruit orientation on seed dispersal. Three groups, pendent- (**a**), horizontal- (**b**) and upward- (**c**) oriented capsules of *Silene chungtienensis*, were used as the material. Each seed deposited on the trap plate has precise coordinates, which were used to analyse the dispersal distance, direction and the general distribution pattern.

**Figure 4:**
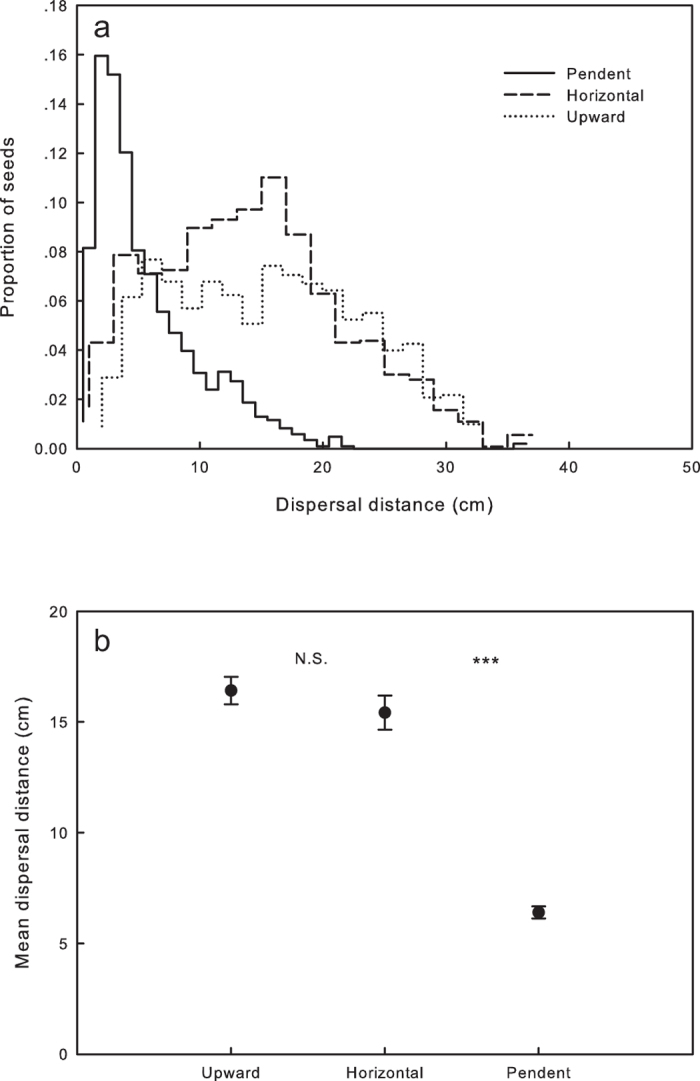
Fruit orientation had a significant effect on the distribution of dispersal distance (**a**) and the mean (±S.E.) dispersal distance (**b**) in *Silene chungtienensis*. Bars indicate standard error. “***” indicates a significant difference at the 0.001 level and “N.S.” indicates a non-significant difference.

To investigate the effects of fruit orientation on seed dispersal, we conducted phenotypic manipulation experiments in Shangri-La Alpine Botanical Garden, between Aug. 10 and Sep. 2 in 2014. We used *Silene chungtienensis* W. W. Smith (Caryophyllaceae) as a model. This plant has pendulous flowers which are pollinated by nocturnal moths, and its stalks become erect after flowering, holding the capsules upward (Fig. 1a). When mature, the seeds are released from an orifice that opens at the apex. One individual produces 1–8 flowers and most of them set fruit which contain 204 seeds on average (178 ~ 286, *N* = 17, weighing ca. 0.15 mg each) under natural conditions, ensuring adequate seeds for experimental study. Natural fruits with stems were harvested just before dehiscence, retaining only the top fruit by cutting the others off. These fruits were then assigned into three groups (Fig. 3): 1) the upward group was intact and the orientation of fruit was kept upward, to provide the control group; 2) the pendent group was manipulated to be down-facing, using a small piece of adhesive tape that do not affect the movement of fruit in the wind; and 3) the horizontal group was manipulated to adopt a horizontal orientation, opening its orifice to the south, again using a small piece of adhesive tape. We chose the southerly direction in order to simulate the possible direction of fruit in wind during the season we studied. To eliminate the effects of plant height on seed dispersal, the stems of these plants were cut so that they were the same height from the base to the centre of the capsule (ca. 21 cm) and were then fixed to a base made of short fine iron wire. This device allows stalks to sway in the wind in the same way as under natural conditions. Each of these plants was set at the centre of a white PVC (polyvinyl chloride polymer, 59.4 × 42.0 cm^2^) plate daubed with Vaseline (to prevent secondary dispersal, which is beyond the scope of this study), modified from Weiblen & Thomson^23^ and Bullock *et al.*^49^. This sticky trap allowed us to observe the distribution of seeds directly on the plates.

#### Dispersal parameters and statistics

Most of the seeds dispersed after five to seven days, and we then captured images of the distribution of seeds on plates using a high resolution digital camera (D7100, Nikon). These images were analysed using PointPicker^50^, a plugin for ImageJ^51^, to obtain the precise coordinates for each seed in units of pixels. We then used these data to calculate the distance from the position of each seed deposited to the mother plant. In order to estimate the direction of seed dispersal, the plate was divided into four quadrants (Fig. 3), and the proportion of seeds in each quadrant was calculated for each fruit sample. The parameters used to estimate dispersal distance (after log-transformation) and direction were compared among the three groups using one-way ANOVA, followed by a Tukey test. In addition, the general pattern of distribution of seeds dispersed from each capsule was estimated with the *G* function (R package spatstat^52^), based on nearest neighbour analysis. This test provides a visualized relationship between the cumulative frequency distribution *G*_*(r)*_and the nearest neighbour distances *r*, representing a general distribution pattern of points (i.e. random, even or cluster). We then used the envelope function (in the spatstat package) to test whether the results differed significantly from complete spatial randomness (CSR). We also estimated the general seed distribution pattern for each group, by pooling the seed coordinates for all samples in that group. Curves from the three groups were plotted on one graph; a steeper (more rapid increase) curve indicates a higher degree of clustering.

## Additional Information

**How to cite this article**: Niu, Y. *et al.* Post-floral Erection of Stalks Provides Insight into the Evolution of Fruit Orientation and Its Effects on Seed Dispersal. *Sci. Rep.* **6**, 20146; doi: 10.1038/srep20146 (2016).

## Supplementary Material

Supplementary Information

## Figures and Tables

**Table 1:** Distribution of species and tests of correlated evolution among fruit orientation and states of three characters from a sub-alpine plant community in SW China.

Character and state	Fruit Orientation		Likelihood ratio
	Upward	Non-upward	
*Function*			19.62^***^
Diaspore	9	18	
Container	44^***^	9	
*Fruit Type*			19.95^***^
Dry	52^***^	15	
Fleshy	1	12	
*Dispersal vector*			22.29^***^
Animal	2	14	
Non-animal	51^***^	13	

^***^P < 0.001.

Number in bold indicates the upward-oriented fruit occurs more frequently in a specific state of each character, examined by Fisher’s exact tests. Likelihood ratio values were calculated from the correlated evolution tests, using the BayesTraits program and the phylogeny based on APG III system.

**Table 2:** Estimates of fixed effects from the Bayesian logistic mixed model that explain the fruit orientation (whether upward or pendent) in a sub-alpine plant community, using phylogeny as a random effect.

Coefficient	Estimate (β)	LCI	UCI	pMCMC
Intercept	3.666	−36.025	41.631	0.842
Function	−27.690	−54.416	−1.608	**0.014**
Type	28.776	−7.263	84.492	0.176
Vector	−2.434	−45.534	32.489	0.968

**Table 3:** One-way ANOVA analysing the proportions of seeds deposited in different directions (quadrants I, II, III and IV, see Fig. 3) on the trap among three groups.

Group	*N*	Quadrants				*F*	*P*
		I	II	III	IV		
Upward	15	0.30^a^	0.23^a^	0.23^a^	0.24^a^	1.512	0.221
Pendent	15	**0.41**^**a**^	0.19^b^	0.15^b^	0.25^ab^	6.225	**0.001**
Horizontal	16	**0.35**^**a**^	0.11^b^	0.17^b^	**0.37**^**a**^	16.964	**<0.001**

Different letters indicates differences at the 0.01 level. *N* indicates sample size.
