# Supplementary material for: Post-floral Erection of Stalks Provides Insight into the Evolution of Fruit Orientation and Its Effects on Seed Dispersal
Source: Sci Rep. 2016 Feb 2;6:20146. doi: 10.1038/srep20146 (PMC4735855; doi:10.1038/srep20146)
Supplement: Supplementary Information [file srep20146-s1.pdf]

Supplementary Information to

Post-floral Erection of Stalks Provides Insight into the Evolution of Fruit Orientation and Its Effects on Seed Dispersal

Yang Niu[1], Zhuo Zhou[1], Wen Sha[1], Hang Sun[1]

[1]Key Laboratory for Plant Diversity and Biogeography of East Asia, Kunming Institute of Botany, Chinese Academy of Sciences, 132 Lanhei Road, 650201, Kunming, Yunnan, China

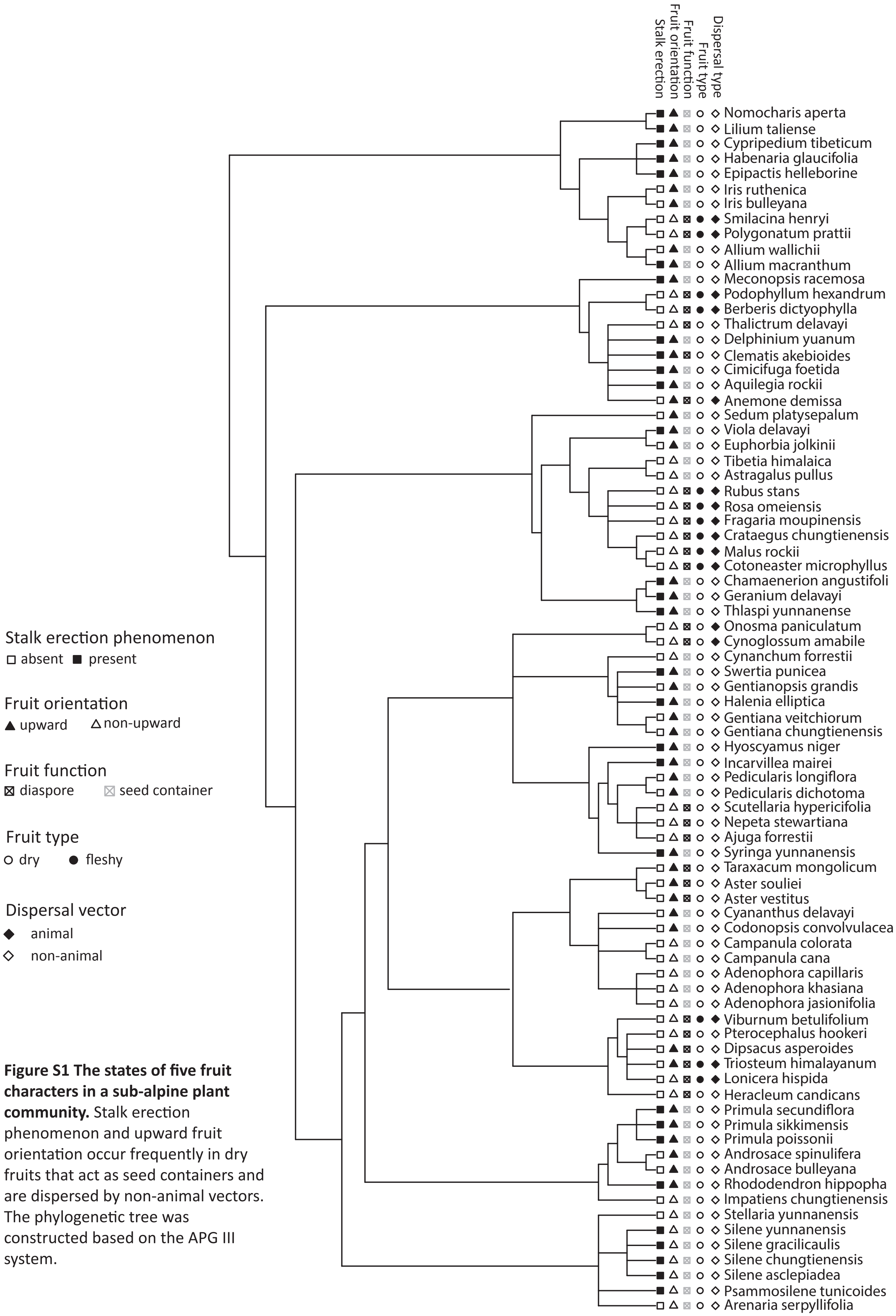

Figure S1 The states of five fruit characters in a sub-alpine plant community. Stalk erection phenomenon and upward fruit orientation occur frequently in dry fruits that act as seed containers and are dispersed by non-animal vectors. The phylogenetic tree was constructed based on the APG III system.

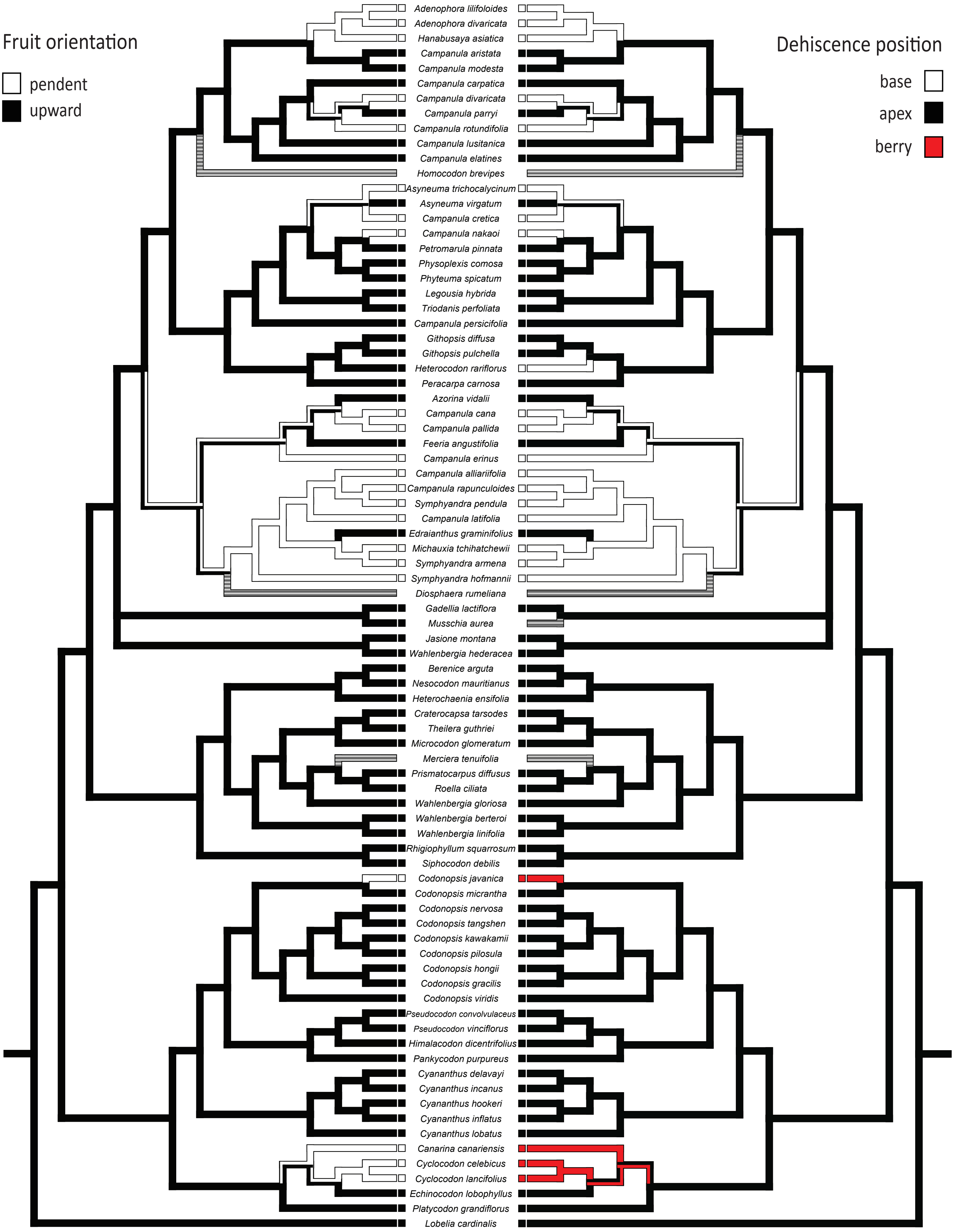

Figure S2 Ancestral character state reconstruction of fruit orientation (left) and dehiscence position (right) in parsimony method based on Bayesian inference tree, and visualized correlation between them in plants of the bluebell family. The tree was reconstructed based on four chloroplast markers (*atpB, matK, rbcL, trnL-trnF*), using 82 taxa representing all the three major clades in the Campanulaceae *s. str*. Note that the cases with unknown dehiscence position or no specific dehiscence position, and cases with indehiscent fruit or berry fruit were treated as missing values.

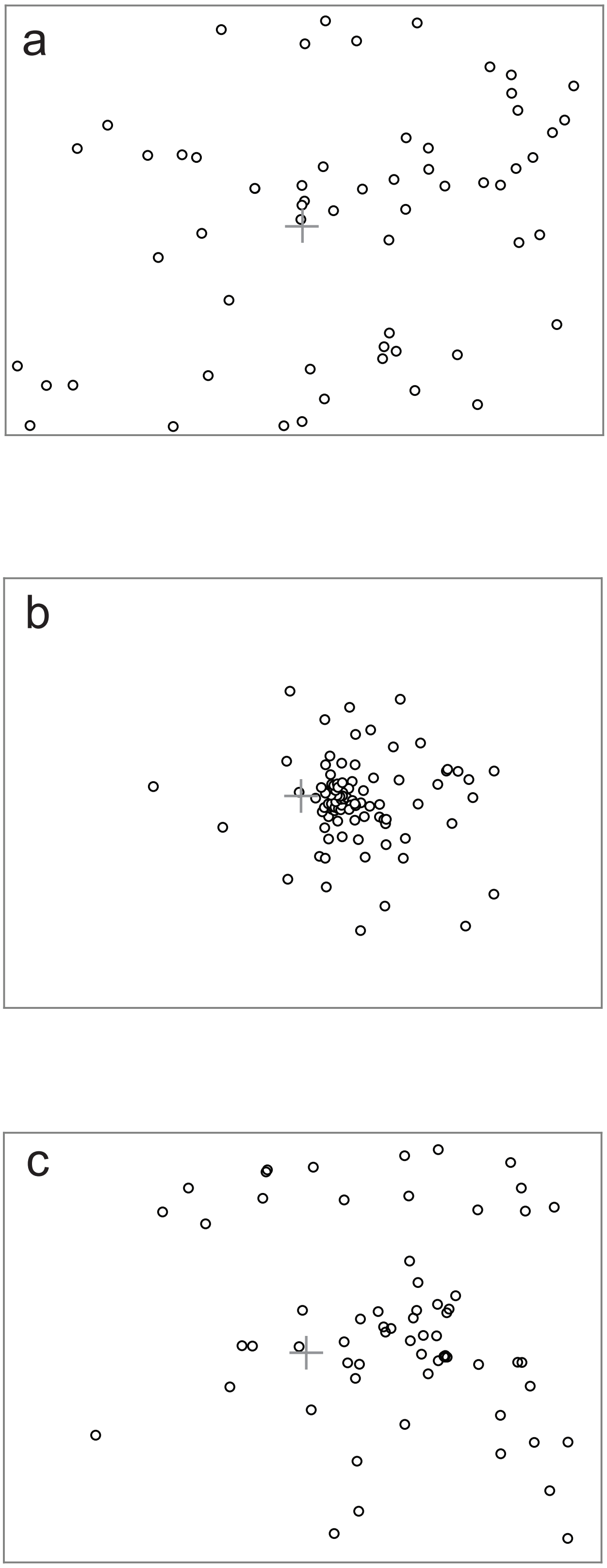

Figure S3 Three cases showing the seeds distribution patterns released from upward- (a), pendent- (b) and horizontal- (c) oriented capsules, respectively.

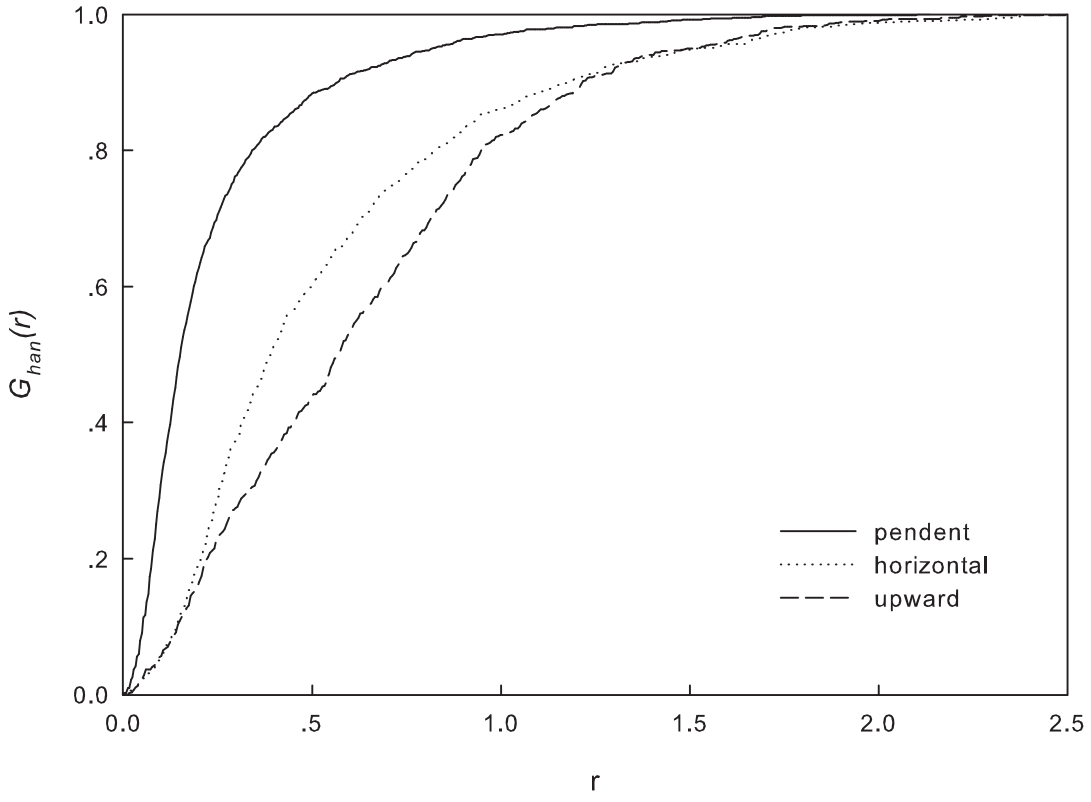

Figure S4 Visualized relationship between the cumulative frequency distribution G(r) and the nearest neighbour distances r, showing the different intensities of clustering in the patterns of the three groups. Note that a steeper (more rapid increase) curve indicates a higher degree of clustering.

Table S1 Distribution of species and tests of correlated evolution among the stalk erection phenomenon and states of three characters from a sub-alpine plant community in SW China. Number in bold indicates the phenomenon that stalks become erect after flowering occurs more frequently in a specific state of each character, examined by Fisher's exact tests. Likelihood ratio values were calculated from correlated evolution tests, using the BayesTraits program and the phylogeny based on APG III system.

	Erection phenomenon		
Character and state	Present	Absent	Likelihood ratio
Function			12.78 **
Diaspore	4	23	
Container	**29*****	24	
Fruit Type			15.13 **
Dry	**33*****	34	
Fleshy	0	13	
Dispersal vector			19.10 ***
Animal	0	16	
Non-animal	**33*****	31	

** $P \leq 0.01$, *** $P < 0.001$

Table S2 Estimates of fixed effects from the Bayesian logistic mixed model that explain the stalk erection phenomenon (present or absent) in a sub-alpine plant community, using phylogeny as a random effect.

Coefficient	Estimate (β)	LCI	UCI	pMCMC
Intercept	-22.170	-45.186	-3.715	**0.010**
Function	-1.528	-8.889	4.973	0.570
Type	-56.162	-110.880	4.575	0.074
Vector	81.392	18.213	129.263	**< 0.001**

Table S3 New sequences for samples of five *Campanula* species used in the study with voucher information and GenBank accession numbers.

Taxon	Voucher	Location	*matK*	*rbcL*	*atpB*	*trnL-F*
Campanula aristata	*ChenYS-1099* (KUN)	China: Sichuan, Xiaojin	KU375231	KU375236	KU375227	KU375241
Campanula cana	*NZ-182* (KUN)	China: Yunnan, Zhongdian	KU375232	KU375237	-	KU375242
Campanula modesta	*SunH-07ZX-0972* (KUN)	China: Xizang, Basu	KU375233	KU375238	KU375228	KU375243
Campanula nakaoi	*NZ-148* (KUN)	China: Xizang, Jilong	KU375234	KU375239	KU375229	KU375244
Campanula pallida	*NZ-130* (KUN)	China: Xizang, Nielamu	KU375235	KU375240	KU375230	KU375245

Table S4 GenBank accession numbers of sequences used in this study for analysis of taxa in the Campanulaceae, with source information.

Taxon	*atpB*	*matK*	*rbcL*	*trnL-F*	Source
Adenophora liliifolioides	JN851154	JN851163	JN851172	JN851181	Zhou et al. 2012
Asyneuma trichocalycinum	-	KC455769	-	FJ426566	Frajman and Schneeweiss, 2009; Schneeweiss et al. 2013
Asyneuma virgatum	EU437665	EU713332	EU713439	-	Haberle et al. 2009
Azorina vidalii	EU437601	EU713266	EU713373	EF088696	Haberle et al. 2009; Roquet et al. 2009
Berenice arguta	EU437622	EU713339	EU713446	-	Haberle et al. 2009
Campanula alliariifolia	EU437604	EU713269	FJ587241	EF088700	Haberle et al. 2009; Roquet et al. 2009
Campanula carpatica	-	EU713303	EU713410	-	Haberle et al. 2009
Campanula cretica	EU437663	EU713330	EU713437	-	Haberle et al. 2009
Campanula dimorphantha	-	-	FJ587246	EF088708	Roquet et al. 2009
Campanula divaricata	EU437676	EU713343	EU713450	EF088718	Haberle et al. 2009; Roquet et al. 2009
Campanula elatines	EU437664	EU713331	EU713438	AJ430970	Haberle et al. 2009; Roquet et al. 2009
Campanula erinus	EU437626	EU713291	EU713398	EF088720	Haberle et al. 2009; Roquet et al. 2009
Campanula latifolia	EU437606	EU713271	EU713378	EF088732	Haberle et al. 2009; Roquet et al. 2009
Campanula lusitanica	EU437667	EU713334	EU713441	EF088733	Haberle et al. 2009; Roquet et al. 2009
Campanula parryi	EU437675	EU713342	EU713449	EF213147	Stefanovic et al. 2008; Haberle et al. 2009
Campanula persicifolia	EU437657	EU713324	EU713431	FJ426573	Frajman et al. 2009; Haberle et al. 2009
Campanula rapunculoides	EU437620	EU713285	EU713392	EF088757	Haberle et al. 2009; Roquet et al. 2009
Campanula rotundifolia	EU437668	EU713336	EU713443	EF213153	Haberle et al. 2009; Roquet et al. 2009
Canarina canariensis	EU437581	EU713246	EU713353	EF088777	Haberle et al. 2009; Roquet et al. 2009
Codonopsis gracilis	JN851161	JN851170	JN851179	-	Zhou et al. 2012
Codonopsis hongii	KC282719	KC282758	KC282788	-	Wang et al. 2013
Codonopsis javanica	KC282685	KC282734	KC282761	-	Wang et al. 2013
Codonopsis kawakamii	KC282697	KC282724	KC282794	-	Wang et al. 2013

Codonopsis micrantha	KC282699	KC282725	KC282795	-	Wang et al. 2013
Codonopsis nervosa	JN851155	JN851164	JN851173	JN851186	Zhou et al. 2012
Codonopsis pilosula	KC282701	KC282743	KC282772	-	Wang et al. 2013
Codonopsis tangshen	JN861156	JN851165	JN851174	JN851187	Zhou et al. 2012
Codonopsis viridis	-	EU713249	EU713356	-	Haberle et al. 2009
Craterocapsa tarsodes	EU437636	EU713301	EU713408	-	Haberle et al. 2009
Cyananthus delavayi	-	KC880529	KC880582	-	Zhou et al. 2013
Cyananthus hookeri	KC880707	KC880546	KC880602	KC880727	Zhou et al. 2013
Cyananthus incanus	KC880708	KC880548	KC880605	KC880728	Zhou et al. 2013
Cyananthus inflatus	KC880711	KC880562	KC880621	KC880731	Zhou et al. 2013
Cyananthus lobatus	KC880713	KC880567	KC880626	KC880733	
Cyclocodon celebicus	KC282716	KC282756	-	-	Wang et al. 2013
Cyclocodon lancifolius	KC282717	KC282757	KC282786	-	Wang et al. 2013
Diosphaera rumeliana	EU437619	EU713284	EU713391	EF088778	Haberle et al. 2009; Roquet et al. 2009
Echinocodon lobophyllus	KC282718	KC282731	KC282787	-	Wang et al. 2013
Edraianthus graminifolius	EU437608	EU713273	EU713380	EF088779	Haberle et al. 2009; Roquet et al. 2009
Feeria angustifolia	EU437622	EU713287	EU713394	EF088780	Haberle et al. 2009; Roquet et al. 2009
Gadellia lactiflora	EU437652	EU713318	EU713425	FJ589212	Haberle et al. 2009; Roquet et al. 2009
Githopsis diffusa	EU437644	EU713310	EU713417	-	Haberle et al. 2009
Githopsis pulchella	EU437647	EU713313	EU713420	-	Haberle et al. 2009
Hanabusaya asiatica	EU437658	EU713325	EU713432	-	Haberle et al. 2009
Heterochaenia ensifolia	EU437666	EU713333	EU713440	-	Haberle et al. 2009
Heterocodon rariflorus	JN851158	JN851167	JN851176	JN851182	Zhou et al. 2012
Himalacodon dicentrifolius	KC282691	KC282736	KC282765	-	Wang et al. 2013
Homocodon brevipes	JN851159	JN851168	JN851177	JN851183	Zhou et al. 2012
Jasione montana	EU437582	EU713247	EU713354	EF088782	Haberle et al. 2009; Roquet et al. 2009

Legousia hybrida	EU437660	EU713327	EU713434	EF088783	Haberle et al. 2009; Roquet et al. 2009
Lobelia cardinalis	EU437598	EU713263	EU713370	DQ356231	Antonelli, 2008; Haberle et al. 2009
Merciera tenuifolia	EU437630	EU713295	EU713402	-	Haberle et al. 2009
Michauxia tchihatchewii	EU437574	EU713239	EU713346	EF088784	Haberle et al. 2009; Roquet et al. 2009
Microcodon glomeratum	EU437627	EU713292	EU713399	-	Haberle et al. 2009
Musschia aurea	EU437638	EU713304	EU713411	EF088785	Haberle et al. 2009; Roquet et al. 2009
Nescodon mauritianus	EU437648	EU773314	EU713421	-	Haberle et al. 2009
Pankycodon purpureus	KC282702	KC282744	KC282773	-	Wang et al. 2013
Peracarpa carnosa	JN851162	JN851171	JN851180	JN851185	Zhou et al. 2012
Peteromarula pinnata	EU437659	EU713326	EU713433	EF088786	Haberle et al. 2009; Roquet et al. 2009
Physoplexis comosa	EU437590	EU713255	EU713362	FJ426586	Frajman and Schneeweiss. 2009; Haberle et al. 2009;
Phyteuma spicatum	EU437589	EU713254	EU713361	EF088787	Haberle et al. 2009; Roquet et al. 2009
Platycodon grandiflorus	EU437586	EU713251	EU713358	EF088788	Haberle et al. 2009; Roquet et al. 2009
Prismatocarpus diffusus	EU437629	EU713294	EU713401	-	Haberle et al. 2009
Pseudocodon convolvulaceus	KC282690	KC282735	KC282764	-	Wang et al. 2013
Pseudocodon vinciflorus	KC282708	KC282730	KC282778	-	Wang et al. 2013
Rhigiophyllum squarrosum	EU437653	EU713319	EU713426	-	Haberle et al. 2009
Roella ciliata	EU437633	EU713298	EU713405	EF088789	Haberle et al. 2009; Roquet et al. 2009
Siphocodon debilis	EU437640	EU713306	EU713413	-	Haberle et al. 2009
Symphyandra armena	EU437611	EU713276	EU713383	EF088704	Haberle et al. 2009; Roquet et al. 2009
Symphyandra hofmannii	EU437605	EU713270	EU713377	GQ254931	Haberle et al. 2009; Stefanovic et al. 2009
Symphyandra pendula	EU437613	EU713278	EU713385	-	Haberle et al. 2009
Theilera guthriei	EU437637	EU713302	EU713409	-	Haberle et al. 2009
Triodanis perfoliata	EU437591	EU713256	EU713363	-	Haberle et al. 2009
Wahlenbergia berteroi	EU437650	EU713316	EU713423	-	Haberle et al. 2009
Wahlenbergia gloriosa	EU437635	EU713300	EU713407	-	Haberle et al. 2009

Wahlenbergia hederacea	EU437628	EU713293	EU713400	EF088792	Haberle et al. 2009; Roquet et al. 2009
Wahlenbergia linifolia	EU437651	EU713317	EU713424	-	Haberle et al. 2009

References

Antonelli, A. 2008. Higher level phylogeny and evolutionary trends in Campanulaceae subfam. Lobelioideae: Molecular signal overshadows morphology. *Molecular Phylogenetics and Evolution* **46**:1-18.

Frajman, B. and G. M. Schneeweiss. 2009. A campanulaceous fate: the Albanian stenoendemic *Asyneuma comosiforme* in fact belongs to isophyllous *Campanula*. *Systematic Botany* **34**:595-601.

Haberle, R. C., A. Dang, T. Lee, C. Peñaflor, H. Cortes-Burns, A. Oestreich, L. Raubeson, N. Cellinese, E. J. Edwards, and S.-T. Kim. 2009. Taxonomic and biogeographic implications of a phylogenetic analysis of the Campanulaceae based on three chloroplast genes. *Taxon* **58**:715-734.

Roquet, C., I. Sanmartín, N. Garcia-Jacas, L. Sáez, A. Susanna, N. Wikström, and J. J. Aldasoro. 2009. Reconstructing the history of Campanulaceae with a Bayesian approach to molecular dating and dispersal–vicariance analyses. *Molecular Phylogenetics and Evolution* **52**:575-587.

Schneeweiss, G. M., C. Pachschwöll, A. Tribsch, P. Schönswetter, M. H. Barfuss, K. Esfeld, H. Weiss-Schneeweiss, and M. Thiv. 2013. Molecular phylogenetic analyses identify Alpine differentiation and dysploid chromosome number changes as major forces for the evolution of the European endemic *Phyteuma* (Campanulaceae). *Molecular Phylogenetics and Evolution* **69**:634-652.

Wang, B. and J. Chen. 2009. Seed size, more than nutrient or tannin content, affects seed caching behavior of a common genus of Old World rodents. *Ecology* **90**:3023-3032.

Zhou, Z., D. Hong, Y. Niu, G. Li, Z. Nie, J. Wen, and H. Sun. 2013. Phylogenetic and biogeographic analyses of the Sino-Himalayan endemic genus *Cyananthus* (Campanulaceae) and implications for the evolution of its sexual system. *Molecular Phylogenetics and Evolution* **68**:482-497.

Table S5 Character information (fruit orientation and dehiscence position) of 82 species from Campanulaceae used in analysis and their sources.

Species	Fruit type	Fruit orientation	Dehiscence position	Source of fruit orientation	Detail	Source of dehiscence position	Detail
Adenophora_lilifoloides	capsule	pendent	base	personal observation	-	publication and personal observation	Lammers 2007
Adenophora_divaricata	capsule	pendent	base	personal observation	-	publication and personal observation	Lammers 2007
Hanabusaya_asiatica	capsule	pendent	base	publication and website	Cullen *et al.* 2011; https://commons.wikimedia.org/wiki/File:Hanabusaya_asiatica_(14420888668).jpg	website	http://pics.davesgarden.com/pics/2008/11/05/altagardener/94787f.jpg
Campanula_aristata	capsule	upward	apex	personal observation	-	publication and personal observation	Hong *et al.* 2011
Campanula_modesta	capsule	upward	apex	personal observation	-	publication and personal observation	Hong *et al.* 2011
Campanula_carpatica	capsule	upward	apex	specimen	http://plants.jstor.org/stable/10.5555/al.ap.specimen.b%20-w%2003796%20-01%200	publication and obanital illustration	Cullen *et al.* 2011
Campanula_divaricata	capsule	pendent	base	specimen	https://plants.ces.ncsu.edu/media/images/Campanula-divaricata--stevbach1--CC-BY.-NC-ND.jpg	pubilication and specimen	Radford *et al.* 1968
Campanula_parryi	capsule	upward	apex	publication	Kearney and Peeble 1942	pubilication	Kearney and Peeble, 1942
Campanula_rotundifolia	capsule	pendent	base	publication	Kearney and Peeble 1942	publication	Kearney and Peeble, 1942
Campanula_lusitanica	capsule	upward	apex	website	http://www.aloj.us.es/carromzar/donyana/VDfotos/VDCampanula_lusitanica.jpg	website	https://es.wikipedia.org/wiki/Campanula_lusitanica
Campanula_elatines	capsule	upward	upper to middle	specimen	http://plants.jstor.org/stable/10.5555/al.ap.specimen.linn-hs309-72-1	pubilication	Cullen *et al.* 2011
Homocodon_brevipes	capsule	unknown	dehiscent by pores or by irregular tears	-	-	publication	Lammers 2007; Hong *et al.* 2011
Campanula_cretica	capsule	pendent	base	website	http://www.flowersofcrete.info/species_list/images/Campanulacretica1.July.jpg	website	http://www.cretanflora.com/campanula_cretica.html
Campanula_nakaoi	capsule	pendent	base	personal observation	-	personal observation	-
Petromarula_pinnata	capsule	upward	upper to middle	website	http://www.alamy.com/stock-photo-flowering-petromarula-	publication	Lammers 2007

					pinnata-cretan-rock-lettuce-a-relative-of-campanula-7488956.html		
Physoplexis_comosa	capsule	upward	apex	website	http://botany.cz/en/physoplexis-comosa/	website	https://it.wikipedia.org/wiki/Physoplexis_comosa
Phyteuma_spicatum	capsule	upward	near apex to middle	website	https://de.wikipedia.org/wiki/Datei:Illustration_Phyteuma_spicatum0.jpg	publication	Lammers 2007
Legousia_hybrida	capsule	upward	apex	website	http://elmer.rbge.org.uk/bgbase/vherb/bgbasevherb.php?cfg=bgbase/vherb/zoom.cfg&filename=E00612918.zip&queryRow=3	botanical illustration	http://plantgenera.org/ILLUSTRATIONS_full_size/154565.jpg
Triodanis_perfoliata	capsule	upward	near apex to middle	website	https://commons.wikimedia.org/wiki/File:Triodanis_perfoliata_biflora_BB-1913.png	publication	Mohlenbrock 1990
Campanula_persicifolia	capsule	upward	apex	botanical illustration	http://plantgenera.org/illustration.php?id_illustration=131817&mobile=0&code_category_taxon=	botanical illustration	http://plantgenera.org/illustration.php?id_illustration=131817&mobile=0&code_category_taxon=
Githopsis_diffusa	capsule	upward	apex	website	http://midwestherbaria.org/imglib/midwest/misc/201504/webmedia_1430104005_web.jpg	publication	Lammers 2007
Githopsis_pulchella	capsule	upward	apex	website	https://commons.wikimedia.org/wiki/File:Githopsis_pulchella_ssp._pulchella.jpeg	publication	Lammers 2007
Heterocodon_rariflorus	capsule	upward	inconspicuous irregular pores near the base	website	http://linnet.geog.ubc.ca/Atlas/Atlas.aspx?sciname=Heterocodon%20rariflorum	website	http://linnet.geog.ubc.ca/Atlas/Atlas.aspx?sciname=Heterocodon%20rariflorum
Peracarpa_carnosa	capsule	pendent	base	personal observation	-	publication	Lammers 2007; Hong *et al.* 2011
Azorina_vidalii	capsule	upward	upper	publication	Tutin *et al.* 1976	publication	Lammers 2007
Campanula_cana	capsule	pendent	base	personal observation	-	personal observation	-
Campanula_pallida	capsule	pendent	base	personal observation	-	personal observation	-
Feeria_angustifolia	capsule	upward	apex	website	http://www.florasilvestre.es/mediterranea/Campanulaceae/Feeria_angustifolia.htm	publication	Lammers 2007

Campanula_erinus	capsule	pendent	base	website	https://commons.wikimedia.org/wiki/File:Campanula_erinus_kz2.JPG	website	https://it.wikipedia.org/wiki/Campanula_erinus
Campanula_alliariifolia	capsule	pendent	base	website	http://www.turkiyebitkileri.com/index.php?dil=en&id=2&familya=14&cins=71&tur=472#.VlF-WNyMV8E	publication	Sell and Murrell 2006
Campanula_rapunculoides	capsule	pendent	base	publication	Stace 1999	publication	Sell and Murrell 2006
Symphyandra_pendula	capsule	pendent	base	website	https://img0.etsystatic.com/058/0/10250940/il_fullxfull.693643450_odx4.jpg	publication	de Candolle, 1830; Cullen *et al.* 2011
Campanula_latifolia	capsule	pendent	base	website	https://en.wikipedia.org/wiki/Campanula_latifolia	publication	http://www.efloras.org/florataxon.aspx?flora_id=5&taxon_id=220002252
Edraianthus_graminifolius	capsule	upward	apex	botanical illustration	http://plantgenera.org/illustration.php?id_illustration=231681&mobile=0&code_category_taxon=	publication	Lammers 2007
Michauxia_tchihatchewii	capsule	pendent	base	publication	Lammers 2007	publication	Lammers 2007
Symphyandra_armena	capsule	pendent	base	website	http://www.biopix.com/zoom.aspx?photoid=81475	publication	de Candolle, 1830; Cullen *et al.* 2011
Symphyandra_hofmannii	capsule	pendent	base	website	https://commons.wikimedia.org/wiki/File:P1000564_Symphyandra_hofmannii_(Campanulaceae)_Plant.JPG	publication	de Candolle, 1830; Cullen *et al.* 2011
Diosphaera_rumeliana	capsule	unknown	unknown	-	-	-	-
Gadellia_lactiflora	capsule	upward	apex	website	http://www.plantarium.ru/page/image/id/265946.html	website	http://www.plantarium.ru/page/image/id/265946.html
Musschia_aurea	capsule	upward	no specific position	botanical illustration	http://plantgenera.org/illustration.php?id_illustration=249767&mobile=0&code_category_taxon=	botanical illustration	http://plantgenera.org/illustration.php?id_illustration=249767&mobile=0&code_category_taxon=
Jasione_montana	capsule	upward	apex	botanical illustration	http://plantgenera.org/illustration.php?id_illustration=131313&mobile=0&code_category_taxon=	publication	Lammers 2007
Wahlenbergia_hederacea	capsule	upward	apex	botanical illustration	https://commons.wikimedia.org/wiki/File:Wahlenbergia_hederacea_Sturm62.jpg	publication	Lammers 2007

Berenice_arguta	capsule	upward	apex	specimen	https://science.mnhn.fr/taxon/species/berenice/arguta	publication	Lammers 2007
Nesocodon_mauritianus	capsule	upward	apex	website	http://img2.iwascoding.com/0/2015/10/13/5E/F69A987575F04206A5178C03CBD7711E.jpg	publication and website	Lammers 2007 http://img2.iwascoding.com/0/2015/10/13/EC/C718ED7E074B48AFB0B76EF50FCB0FF0.jpg
Heterochaenia_ensifolia	capsule	upward	apex	specimen	http://plants.jstor.org/stable/10.5555/al.ap.specimen.g00314630	publication	Lammers 2007
Craterocapsa_tarsodes	capsule	upward	apex	specimen	http://plants.jstor.org/compilation/Craterocapsa.tarsodes	specimen	http://plants.jstor.org/compilation/Craterocapsa.tarsodes
Theilera_guthriei	capsule	upward	apex	specimen	http://plants.jstor.org/stable/10.5555/al.ap.specimen.k000425211	publication	Lammers 2007
Microcodon_glomeratum	capsule	upward	apex	specimen	http://plants.jstor.org/stable/10.5555/al.ap.specimen.tcd0003320	publication	Lammers 2007
Merciera_tenuifolia	capsule	unknown	indehiscent	-	-	publication	Lammers 2007
Prismatocarpus_diffusus	capsule	upward	apex	specimen	http://plants.jstor.org/stable/10.5555/al.ap.specimen.m0185303	publication	Lammers 2007
Roella_ciliata	capsule	upward	apex	specimen	http://plants.jstor.org/stable/10.5555/al.ap.specimen.bm000925023	publication	Lammers 2007
Wahlenbergia_gloriosa	capsule	upward	apex	website	http://www.rbgsyd.nsw.gov.au/__data/assets/image/0004/79879/Wahlenbergia_gracilis_fruit_620.JPG	publication	Lammers 2007
Wahlenbergia_berteroi	capsule	upward	apex	botanical illustration	http://plantgenera.org/illustration.php?id_illustration=80031&mobile=0&code_category_taxon=	publication and specimen	Lammers 2007
Wahlenbergia_linifolia	capsule	upward	apex	specimen	http://plants.jstor.org/stable/10.5555/al.ap.specimen.g00314590	publication and botanical illustration	Lammers 2007
Rhigiophyllum_squarrosum	capsule	upward	upper, circumscissile	specimen	http://plants.jstor.org/stable/10.5555/al.ap.specimen.pre0190274-0	publication	Lammers 2007

Siphocodon_debilis	capsule	upward	upper, circumscissile	specimen	http://plants.jstor.org/stable/10.5555/al.ap.specimen.bol138696	publication	Lammers 2007
Cononopsis_javanica	berry	pendent	-	-	-	-	-
Codonopsis_micrantha	capsule	upward	apex	personal observation	-	personal observation	-
Codonopsis_nervosa	capsule	upward	apex	personal observation	-	publication and personal observation	Hong et al. 2011
Codonopsis_tangshen	capsule	upward	apex	personal observation	-	publication and personal observation	Hong et al. 2011
Codonopsis_kawakamii	capsule	upward	apex	personal observation	-	publication and personal observation	Hong et al. 2011
Codonopsis_pilosula	capsule	upward	apex	website	http://bruce0342.blogspot.com/2008_09_01_archive.html	publication	Lammers 2007
Codonopsis_hongii	capsule	upward	apex	personal observation	-	personal observation	-
Codonopsis_gracilis	capsule	upward	apex	personal observation	-	personal observation	-
Codonopsis_viridis	capsule	upward	apex	personal observation	-	personal observation	-
Pseudocodonopsis_convolvulaceus	capsule	upward	apex	specimen	in KUN and PE	Publication	Hong et al., 2011
Pseudocodonopsis_vinciflorus	capsule	upward	apex	speciemen	in KUN and PE	personal observation	-
Himalacodon_dicentrifolius	capsule	upward	apex	specimen	in KUN and PE	publication and specimen	Hong et al., 2011
Pankycodon_purpureus	capsule	upward	apex	specimen	in KUN and PE	publication and specimen	Hong et al., 2011
Cyananthus_delavayi	capsule	upward	apex	personal observation	-	personal observation	-
Cyananthus_incanus	capsule	upward	apex	personal observation	-	personal observation	-
Cyananthus_hookeri	capsule	upward	apex	personal observation	-	personal observation	-
Cyananthus_inflatus	capsule	upward	apex	personal observation	-	personal observation	-
Cyananthus_lobatus	capsule	upward	apex	personal observation	-	personal observation	-
Canarina_canariensis	berry	pendent	-	website	https://commons.wikimedia.org/wiki/File:Bic%C3%A1caro_(Canarina_canariensis).JPG	publication	Lammers 2007
Cyclocodon_celebicus	berry	pendent	-	personal observation	-	publication	Hong *et al.* 2011
Cyclocodon_lancifolius	berry	pendent	-	personal observation	-	publication	Hong *et al.* 2011

Echinocodon_lobophyllus	capsule	upward	apex	specimen	http://www.cvh.org.cn/spm/PE/01264248	specimen	http://www.cvh.org.cn/spm/PE/01264248
Platycodon_grandiflorus	capsule	upward	apex	personal observation	-	personal observation	-
Lobelia_cardinalis	capsule	upward	apex	website	http://castle.eiu.edu/~prairie/images/lobecard.fruits1.jpg	publication	Lammers 2007
Asyneuma_trichocalycinum	capsule	pendent	base	website	http://www.actaplantarum.org/acta/galleria1.php?aid=1595	publication	Lakušić and Conti 2004 and references therein
Asyneuma_virgatum	capsule	upward	apex	website	http://vanherbaryum.yyu.edu.tr/flora/azortandir/asyneumavivi/pages/Asyneuma%20virgatum%20%20subsp_%20%20virgatum_jpg.htm	website	http://www.turkiyebitkileri.com/index.php?dil=tr&id=2&familya=14&cins=70&tur=3060#.VlKHG9yMV8E

References

Cullen, J., Knees, S.G., Cubey, H.S. (2011) *The European garden flora flowering plants: a manual for the identification of plants cultivated in Europe, both out-of-doors and under glass* Cambridge University Press.

De Candolle, A. (1830) *Monographie des campanulées* Veuve Desray.

Hong, D., Ma, L., Ge, S., Lammers, T. & Klein, L.(2011) Campanulaceae. In *Flora or China*, Vol. 19 (eds Wu, Z.-Y. et al.) 505-563 Missouri Botanical Garden Press and Science Press.

Kearney, T.H. and Peebles, R.H. (1942) *Flowering plants and ferns of Arizona* US Dept. of Agriculture.

Lakušić, D. and Conti, F. (2004) *Asyneuma pichleri* (Campanulaceae), a neglected species of the Balkan Peninsula. *Plant Systematics and Evolution*, 247, 23-36.

Lammers, T. (2007) Campanulaceae. In *Flowering Plants· Eudicots*, Springer: pp 26-56.

Mohlenbrock, R.H. (1990) *Flowering Plants: Nightshades to Mistletoe* SIU Press.

Radford, A.E., Ahles, H.E., Bell, C.R. (1968) *Manual of the vascular flora of the Carolinas* University of North Carolina Press.

Sell, P. and Murrell, G. (2006) *Flora of Great Britain and Ireland: Volume 4, Campanulaceae-Asteraceae* Cambridge University Press.

Stace, C.A. (1999) *Field flora of the British Isles* Cambridge University Press.

Tutin, T., Heywood, V., Burges, N., Moore, D., Valentine, D., Walters, S., Webb, D. (1976) *Flora Europaea: Plantaginaceae to Compositae (and Rubiaceae). Vol. 4* Cambridge: University Press.
